# A scoping review exploring reablement models of training and client assessment for older people in primary health care

**DOI:** 10.1017/S1463423621000918

**Published:** 2022-02-24

**Authors:** Marguerite Bramble, Sarah Young, Sarah Prior, Hazel Maxwell, Steve Campbell, Annette Marlow, Douglass Doherty

**Affiliations:** 1 Charles Sturt University, School of Nursing, Paramedicine and Healthcare Sciences, Bathurst, 2795, NSW, Australia; 2 University of Tasmania, College of Health and Medicine, Newnham Campus, Launceston, TAS, 7250, Australia; 3 Tasmanian School of Medicine, Cradle Coast Campus, Burnie, TAS, 7320 Australia; 4 University of Tasmania, School of Health Sciences, Rozelle Campus, Sydney, NSW, Australia; 5 University of Tasmania, School of Nursing, College of Health and Medicine, Newnham Campus, Launceston, TAS, 7250 Australia; 6 Family Based Care Tasmania, Chief Executive Officer, Family Based Care Tasmania, Burnie, TAS, 7320 Australia

**Keywords:** assessment, community, frameworks, interdisciplinary, reablement, training

## Abstract

**Aim:**

The aim of this scoping review is to explore the evidence by which community service providers have integrated reablement models of staff training and client assessment into practice.

**Background:**

The concept of reablement, which has emerged during the last two decades globally, has recently been defined by health experts from 11 countries through a Delphi study. Reablement is seen as a way to support integrated frameworks that achieve person-centred, long-term care and assistance across community settings. International research indicates there is some evidence of developing models of reablement that include staff training and individual components of client assessment. However, evidence of integrating reablement into interdisciplinary practice continues to be sparse.

**Methods:**

The review adopted the preferred Reporting Items for Systematic Reviews and Meta-Analyses Extension for Scoping Reviews (PRISMA-ScR) approach. Inclusion criteria for the review related to community care, primary care, long-term care, and residential care. Populations of interest included service providers, interdisciplinary staff, trainers, and assessors.

**Results:**

A total of 11 papers were reviewed. The studies varied in their approach to reablement training and client assessment frameworks. Three studies included assessment of staff well-being. All included evidence-based, person-centred components that can be integrated across health care settings. Single disciplinary approaches were used in all studies and some included training evaluation.

**Conclusion:**

This review has identified that currently reablement models are not yet embedded as frameworks for practice by community service providers in primary health care settings. Different programmes of training and assessment are being designed based on single disciplinary approaches and the context in which they are delivered. Further developmental work is required to integrate the components of discipline-specific training programmes within interdisciplinary frameworks. This will achieve not only an integrated framework for delivery across settings but also further the success of ‘ageing in place’ policy.

## Introduction

The pace of population ageing worldwide is increasing and by 2050 it is expected to reach 2 billion, up from 15 million in 2015 (World Health Organisation (WHO), 2017). By 2021 and beyond, the number of people 60 years and older will outnumber children younger than five years. With this demographic shift comes the challenges to ensure health and social systems can provide environments to support extra years of life by living independently and in relatively good health (WHO, 2017). The concept of healthy ageing supports the ‘ageing in place’ policy, where older people can continue to live at home or within their communities despite significant declines in capacity. This shift in focus centres new models of care firmly within primary health care principles and interdisciplinary community services that can promote dignity, autonomy, functioning and continued personal growth (AIHW, 2016; 2020).

Healthy ageing is defined by the WHO as ‘the process of developing and maintaining the functional ability that enables well-being in older age’ (WHO, 2017:12). Healthy ageing takes into account that even when chronic diseases do emerge, their consequences can be limited through high-quality and integrated primary health care to strengthen and maintain capacity or reverse decline (Smith *et al*., 2016; Kluge *et al*., 2018). In countries with more developed primary health care systems, strategies have been initiated to promote multi-sectoral action supporting integrated frameworks to achieve person-centred, long-term care in community settings (Metzelthin *et al*., 2020). Similarly in Australia, state and commonwealth funded integrated care services are developing that cross the boundaries between primary, community, acute health, and social care settings to support screening and assessment pathways for conditions such as dementia (Alzheimer’s Australia, 2015). However, to date, similar evidence-informed models of practice that include staff training and interdisciplinary client assessment pathways to support capacity and overall wellness in the community for older people continue to be sparse (Australian Association of Gerontology, 2020).

One response to the need for a more inclusive framework is the concept of reablement, which has emerged during the last two decades both within Australia and globally, and has recently been defined through a Delphi study comprising of reablement experts from 11 countries (Metzelthin *et al*., 2020:11). There has been some debate internationally about the differences between reablement, rehabilitation, and restorative programmes based on definitions by the World Health Organisation (WHO) and the Department of Health in the UK (Legg *et al*., 2016). The current consensus definition below addresses this debate by clearly describing reablement as a programme focused on home-based rather than institutionalised or hospital care.

The key concepts as captured by the current consensus definition of reablement are as follows:

*Reablement is a person-centred, holistic approach that aims to enhance an individual’s physical and/ or other functioning …. Reablement … is delivered by a trained and coordinated interdisciplinary team. Reablement supports an individual to achieve their goals, if applicable, through participation in daily activities, home modifications and assistive devices as well as involvement of their social network. Reablement is an inclusive approach irrespective of age, capacity, diagnosis or setting.* (Metzelthin *et al*., 2020:11)

The concept of reablement was first adopted in Australia in the early 2000s in the state of Western Australia. Since this time, the practice of ‘wellness and reablement’ as it is termed in Australia, has grown nationwide but is still a relatively new model for providing services in primary health care settings, with very little evidence of workforce development (Australian Association of Gerontology, 2020). Systematic reviews to date, mainly from the United Kingdom and Scandinavian countries, have focussed on the outcomes of using reablement in community and other social care environments, with limited focus on staff training, such as for support workers, assistant workers, and allied health professionals (Moran *et al*., 2015). Some studies have identified that future research should focus on evaluating the implementation of agreed reablement components to inform practice, education, and policy (Laragy and Allen, 2015; WA Home and Community Services, 2019).

Reablement interventions and activities are designed collaboratively with clients to focus generally on activities of daily living with the aim of allowing people to ‘age in place’ in their own residence and participate socially as they wish. Programmes are delivered predominantly by community service providers who focus on personalised health and support to enable people to continue living in their homes safely and independently.

There is some evidence that embedding reablement as an organisational framework, or model of care in practice, can be problematic as opportunities for staff education and training are not yet well established in Australia (Maxwell *et al*., 2021). One innovative training programme adopted recently by a community service provider demonstrated the importance staff placed on client-centred care and working in partnership with clients to achieve goals following the training (Maxwell *et al*., 2021). Reablement principles were applied and embedded in the creation and development of the learning and teaching materials, the teaching programme as well as the evaluation of the impact of reablement across the organisation (Maxwell *et al*., 2021). However, the outcomes from this and other programmes have been limited in developing robust models of training and client assessment that could be embedded in practice across disciplines in primary health care.

## Research aim

The aims of this scoping review are

1. To explore the evidence by which a reablement framework has been integrated by community service providers into existing models of training and client assessment; and
2. To explore the methods for evaluation of reablement training programmes in practice.

## Methods

This scoping review was designed with the intention of exploring to what extent there is evidence that a reablement framework has been integrated by service providers into existing models of training and assessment. To achieve this aim, a literature review method was adopted, using the Preferred Reporting Items for Systematic Reviews and Meta-Analyses Extension for Scoping Reviews (PRISMA-ScR) approach (Moher *et al*., 2009; Peters *et al*., 2015; Tricco *et al*., 2018), (see Table 1). For the purposes of the scoping review, papers including training programmes were limited to those that by definition focused on reablement and to client assessment that focused on positive outcomes. In accordance with PRISMA-ScR stages 12 and 13, individual papers were then critically assessed using these definitions of training and client assessment, then synthesised and summarised (see Table 2).

**Table 1.** Search concepts and key search terms

	Search Concept	Key words and Boolean terms
#1	Reablement services	“reablement” AND [“long-term care” OR “long-term services”] AND “interdisciplin*” AND “residen*” AND “function*” and “independ*”, AND [“community” OR “primary”]
#2	Integration to training and assessment	“reablement” AND “model” AND [“train*” OR “assess*”]
#3	Evidence of sustainability	“reablement” AND “sustainab*” AND [“model” OR “framework”]

**Table 2.** Characteristics and findings of included studies of reablement training and client assessment

First author & year & country of origin	Study population	Intervention type	Study aims	Methodology	Outcome measure	Important results (training regime & outcomes)
Bergstrom *et al*. (2019) Sweden	Older community dwelling persons; home care staff delivering ASSIST	Protocol for ASSIST 1.0 (a theory-based reablement program) feasibility	Feasibility study of the intervention (ASSIST 1.0)	Mixed methods	The main outcome measure of the proposed intervention is the degree of change in the older persons’ perception of their performance in the activities addressed by the intervention.	The proposed intervention has a workshop component and coaching sessions which occur weekly for a minimum of 10 weeks. As this is a protocol for a feasibility study, the outcomes have not yet been determined, but will form the basis for refining the ASSIST 1.0 program.
Eliassen *et al*. (2020) Norway	Physiotherapists (PTs), home trainers (HTs) & reablement recipients	Integration of physiotherapy into user-centred service delivery	Explore how physiotherapy practice is performed in reablement settings and the content of the service.	Qualitative	Division of labour, assessment, and content of reablement intervention.	The importance of responsivity associated with higher flexibility of labour division between team members (PTs & HTs) in the reablement process; efficiency associated with nonspecific approach.
Gerrish *et al*. (2017) United Kingdom	Patients with chronic conditions	Medicines reablement	Evaluate success of training package to reable patients following transition from hospital.	Mixed methods	Assessment of patient independence (patients), training package (staff).	Pharmacy technicians delivered a half-day training programme to other support workers in medicines reablement. Increased patient independence and staff satisfaction resulted from this programme.
Gustafsson *et al*. (2019) Sweden	Older people after a period in hospital	Multi-professional team caring skills	Illuminate older persons’ perceptions of as success factors for health support in reablement.	Qualitative	Success factors for caregiving.	Existing nursing staff were given a 1-month training programme to improve skills prior to the intervention (a 3-month in-home reablement programme). Most important traits in caregivers identified and recommended to be included in education/training of future caregivers.
Lawn *et al*. (2017) Australia	Support workers (SWs) & coordinators; client group older people	SWs & coordinators training program	Enhance knowledge, skills, and confidence of community aged care SWs.	Quantitative survey	Training outcomes; SWs and coordinators; collaboration.	Training was a one-day workshop. Participants reported improved confidence, knowledge & skills; improved SWs’ ability to foster a cooperative approach to supporting clients.
Low *et al*. (2018) Australia	Residential care staff & residents	Lifeful – 12 month reablement training programme in residential care	Feasibility study of Lifeful reablement program	Mixed methods	Job satisfaction, resident outcomes.	A reablement training programme was provided at aged care facilities with the training composed of four x 3hr sessions over 12 months. Staff found the programme ‘acceptable’ but had suggestions for improvement. Residents showed some improvements with the programme, including reduction in depression symptoms.
Metzelthin *et al*. (2017) Netherlands	Older community dwelling adults	Stay Active at Home (SaaH) Program	Detail rationale & content of Stay Active at Home Program	Qualitative review	Effectiveness of the proposed intervention based on an established checklist (‘Template for Intervention Description [TIDieR]’).	This study used an established tool (TIDieR) to show that SaaH is a comprehensive training programme for home care staff to deliver day-to-day services at home from a reablement perspective.
Resnick *et al*. (2009) USA	Residential care nursing assistants (NAs) & residents	12-month restorative care (‘Res-Care’) intervention	Test effectiveness of Res-Care intervention	Randomised Controlled Trial	Quantitative assessment of NAs’ knowledge, changing care behaviours, performance of Res-care, self-efficacy & expectations.	NAs showed increased understanding of and implementation of res-care activities.
Ritchie *et al*. (2017) United Kingdom	Hospitalised older adults	Restorative care training package administered to nursing assistants (NAs) in two community subacute care wards	Increase restorative care interactions between NAs and clients	Quantitative prospective cohort study	Frequency of NA-led restorative care events.	Training was combination of didactic teaching and hands-on training on the ward. NA-led restorative care events increased significantly after training was conducted.
Smeets *et al*. (2019) Netherlands	Home-care workers	Stay Active at Home Program (SaaH)	Explore the experience of home-care workers applying a reablement programme	Qualitative	Knowledge, skills, self-efficiency, social support.	Experiences of home-care workers with the ‘SaaH’ Programme were assessed. It was found to be useful to apply reablement practices, but that more support was needed to master skills & deal with challenging situations.
Zingmark *et al*. (2019) Sweden	Physiotherapists (PTs) & Occupational therapists (OTs)	OT & PT interventions	Critical reflection on practice	Quantitative assessment of reablement interventions	Focus, content, duration of reablement interventions.	Critically reflect on focus, content and duration of different reablement interventions; importance of using valid & reliable assessments to evaluate effects.

In a scoping review, it is important to establish inclusion criteria to determine which studies are eligible for inclusion in the review (Peters *et al*., 2015). In this case, the context of included studies was those related to community care, primary care, long-term care, and residential care. The populations of interest included service providers, interdisciplinary staff, trainers, patients/clients, and assessors. Studies were restricted to English language with a date range of 2009–2020. Peer-reviewed literature, as well as grey literature and dissertations, was eligible for inclusion in the study.

## Search strategy

**Figure 1.**
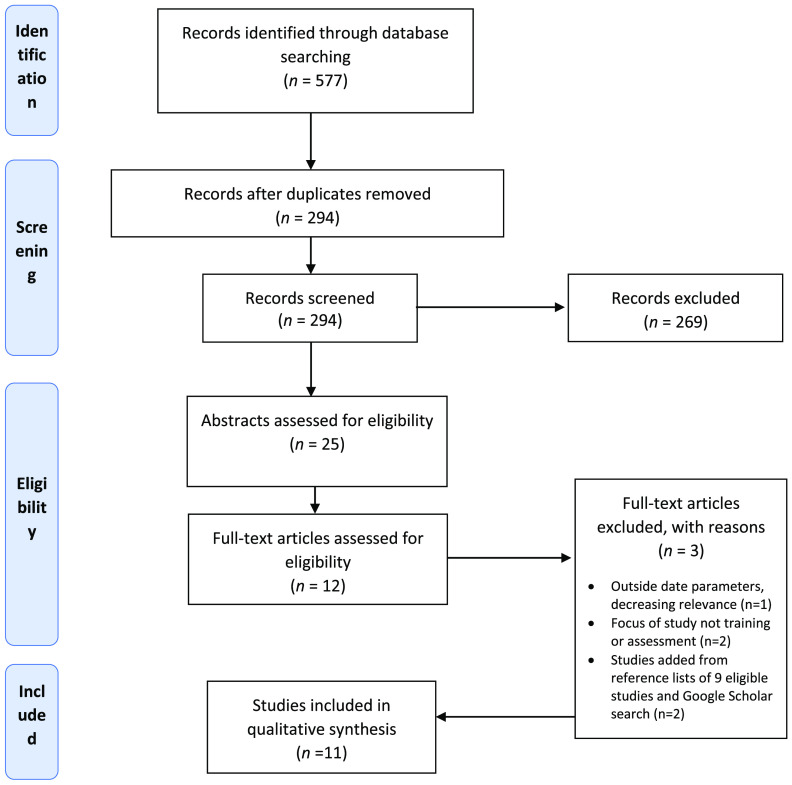
PRISMA flow diagram for the scoping review process

The search strategy (Figure 1) used a combination of terms based on three concepts using electronic databases JBI, Scopus, CINAHL, PsycInfo, Web of Science, Medline & Embase via OVID, Psychology & Behavioural Sciences collection, and Google Scholar. A Boolean search was conducted using key terms developed from the search concepts (Table 1).

From the seven databases, 577 results were retrieved and exported to EndNote®. After automatic and manual removal of duplicates, 294 records remained. The titles and abstracts of these records were assessed against the inclusion criteria listed above. Of the 294 records assessed, 25 were retained for abstract review. An online software platform, Covidence®, was used to facilitate this review process. All abstracts and metadata were imported into Covidence® to facilitate the implementation of the PRISMA-ScR screening approach (see Figure 1). Of the 12 articles remaining after abstract screening, three were excluded after full-text review. Two further studies were added after reviewing the reference lists of the nine eligible articles and conducting a Google Scholar search for completeness, for a total of 11 articles included in this review. A single author (SY) screening process was used for the title/abstract screening, and three authors (SY, SP, MB) participated in the full-text review process. The validity of the papers included in the full-text review was assessed against the eligibility criteria:

- The paper relating to reablement in the context of community service provision; and
- The paper making specific reference to reablement as a component of training or assessment.

If there was an affirmative answer to both questions, the paper was retained. One article (Ritchie *et al*., 2017) was retained despite its hospital setting because of the potential the hospital-based reablement interventions described could impact patient engagement with reablement care post-discharge in the community. Consequently, the utility of the insights provided by the study in terms of future reablement programme design was deemed valuable. Papers were assessed for quality as part of the review process. All nine included papers were appraised to ensure methodological rigor during the full-text review stage (by three authors – SY, SP, MB).

## Data extraction and charting

For each article, the author, year, study population, country of origin, intervention type, study aims, methodology, outcome measure(s), and important results were abstracted from the article, as per guidance for scoping review procedures (Arksey & O’Malley, 2005; Peters *et al*., 2015). A summary of these findings is shown in Table 2. The findings will be discussed in narrative format in combination with discussion of these results in the following sections.

## Results

Three qualitative papers, four quantitative, and four mixed-method studies were identified (Table 2). Eight of the 11^1^ studies included in this review provided discussion of staff training regimes for reablement care in a community setting, with the exception of one study that was conducted in a hospital setting (Ritchie *et al*., 2017). Four of the reablement training programmes comprised an educational, theoretical component in combination with a more hands-on or practical component (Resnick *et al*., 2009; Ritchie *et al*., 2017; Low *et al*., 2018; Bergstrom *et al*., 2019). Four other studies included an educational component with a role-play or simulation element added (Gerrish *et al*., 2017; Lawn *et al*. 2017; Metzelthin *et al*., 2017; Smeets *et al*., 2019). A ‘student-centred’ approach with consideration of adult learning styles, inclusion of interactive components and open discussion with reduced focus on ‘lecturing’, was described in two of the training programmes (Lawn *et al*., 2017; Ritchie *et al*., 2017). Case studies were used as teaching tools in two studies, and these same two studies provided course manuals for their enrollees (Lawn *et al*., 2017; Ritchie *et al*., 2017).

### Study population

In terms of staff reablement training programme delivery, a variety of different disciplines delivered the training across the eight studies, including an occupational therapist (Bergstrom *et al*., 2019), members of the research team conducting the study (Lawn *et al*., 2017; Low *et al*., 2018), nurses with varying levels of education and capability (Resnick *et al*., 2009), physiotherapists (Gerrish *et al*., 2017; Ritchie *et al*., 2017), or a combination of nurses and research team members (Metzelthin *et al*., 2017; Smeets *et al*., 2019). In all cases, the programmes were being delivered to staff responsible for client care including rehabilitation assistants and support workers (Gerrish *et al*., 2017); community support workers and their supervisors (Lawn *et al*., 2017); nursing assistants (Ritchie *et al*., 2017); home care staff (Low *et al*., 2018; Bergstrom *et al*., 2019); nurses and domestic support workers (Metzelthin *et al*., 2017; Smeets *et al*., 2019); and nurses, nursing assistants, and other interested care staff (Resnick *et al*., 2009).

### Theoretical frameworks

With regard to the design of the reablement training programmes, many of the programmes described an explicit theoretical underpinning, such as Bandura’s theory of self-efficacy and behaviour change (Resnick *et al*., 2009; Metzelthin *et al*., 2017; Smeets *et al*., 2019), or a combination of Bandura’s social learning theory and Kotter’s eight-step model for change (Low *et al*., 2018). The role of behaviour change and motivation in reablement care, without specific reference to Bandura’s theories, was also referred to by one programme (Lawn *et al*., 2017). Most programmes also mentioned adopting a person-centred approach to care (Metzelthin *et al*., 2017; Low *et al*., 2018; Bergstrom *et al*., 2019), or the importance of person-centred care (Lawn *et al*., 2017).

In reference to the detailed content of the reablement training programmes, the more educational and theoretical elements of the programmes had a variety of foci. Common topics for teaching included: an introduction to or setting the context for reablement and reablement theory (Resnick *et al*., 2009; Gerrish *et al*., 2017; Low *et al*., 2018; Bergstrom *et al*., 2019); strategies for practical care activities or interventions (e.g., exercise, bathing, transfers, and medication administration) (Resnick *et al*., 2009; Gerrish *et al*., 2017; Low *et al*., 2018); encouraging and motivating older adults (Resnick *et al*. 2009; Lawn *et al*., 2017; Metzelthin *et al*., 2017; Smeets *et al*., 2019); and incorporating reablement activities into existing care regimes (Resnick *et al*., 2009).

### Practical components

The practical components included in three of the training programmes (Resnick *et al*., 2009; Ritchie *et al*., 2017; Bergstrom *et al*., 2019) were quite varied. In the Bergstrom *et al*. (2019) study, the practical elements were focused on the priorities of the older person involved in the study. The occupational therapist (OT) providing the training worked together with the staff and older person to facilitate reablement for a particular task identified by the client as important to them (e.g., independent grocery shopping), and reablement for this task, including site visits if needed, became the focus (Bergstrom *et al*., 2019). In the Resnick *et al*. (2009) study, the practical component was focused on the work of a ‘champion’ for the reablement programme, a role filled by a ‘Res-Care’ Nurse Coordinator (RCN) from the research team. The RCN helped the staff engage with the residents in reablement-focused activities and provided on-going practical learning opportunities for the staff participating in the training programme (Resnick *et al*., 2009). Lastly, in the Ritchie *et al*. (2017) study, the physiotherapist worked with each staff member in the training programme in ward-based practice sessions to reinforce reablement principles and to help identify reablement opportunities.

Four studies (Gerrish *et al*., 2017; Lawn *et al*. 2017; Metzelthin *et al*., 2017; Smeets *et al*., 2019) included ‘role play’ or ‘simulation’ activities as a form of practical component in their training programmes. In the Gerrish *et al*. (2017) study, the competencies of the staff being trained in medicines reablement were assessed by the physiotherapists training them through simulation and role-play activities. In the Lawn *et al*. (2017) study, video clips were used to demonstrate skills in reablement practice, after which role-play was used to put these ideas into action. In the ‘Stay Active at Home’ program described by both Metzelthin *et al*. (2017) and Smeets *et al*. (2019), practical assignments/tasks were given between didactic training sessions to simulate the application of skills in practice.

### Staff training evaluation

In the studies where reablement training programmes were administered as part of the research, in some cases evaluation of the training programmes was conducted (Table 1). In four cases (Lawn *et al*., 2017; Ritchie *et al*., 2017; Low *et al*., 2018; Smeets *et al*., 2019), this training programme evaluation was explicitly undertaken and reported, and in two other cases the evaluation was implicit in the reporting of the findings (Resnick *et al*., 2009; Gerrish *et al*., 2017).

In the Lawn *et al*. (2017) study, 102 of the 140 trainees completed the programme evaluation survey, and the ‘majority’ (56.4%–76.6%) agreed that the training programme had improved their ‘confidence, knowledge, and skills’, and they better understood the principles of how to support clients in reablement-orientated behaviour change (p. 461). In the Low *et al*. (2018) study, 104 staff members provided an evaluation of the training programme. The majority (99.3%) of staff found the training material easy to understand, that it improved their understanding of the concepts presented, and ‘helped them build better relationships with residents’ (p. 5). In the Ritchie *et al*. (2017) study, evaluation of the reablement training programme was undertaken using observational measurements of the frequency of reablement care events pre- and post-intervention. A statistically significant proportional increase (74% to 92%) in these events was documented over the course of the study (Ritchie *et al*., 2017). Smeets *et al*. (2019) found the trainees in their study perceived the ‘Stay Active at Home’ reablement training programme as useful for the application of reablement, and found the programme helpful in reminding them to apply reablement principles in their care practice.

While the Resnick *et al*. (2009) study did not explicitly conduct an evaluation of their reablement training programme, the outcome measures could be considered an evaluation in terms of programme effectiveness. The nursing assistants (NAs) who completed the programme described having increased knowledge of and beliefs about the benefits of reablement care after 12 months in the programme than the NAs in the control group (Resnick *et al*., 2009). However, it was noted that the frequency of reablement care events did not increase over the 12 months. In the Gerrish *et al*. (2017) study, the trainees found the reablement training ‘informative’ and ‘highly relevant’, and that they had gained the skills necessary to support patients in medicines reablement (p. 308).

While the reablement training was evaluated as the only outcome measure in four studies (Resnick *et al*., 2009; Lawn *et al*., 2017; Ritchie *et al*., 2017; Smeets *et al*., 2019), in two studies (Gerrish *et al*., 2017; Low *et al*., 2018), the outcome measures included both the reablement training evaluation and a measure of the reablement outcomes, for example assessment of clients. One study (Bergstrom *et al*., 2019) included client capabilities as the only outcome measure.

### Client assessment

The reablement programme evaluation outcomes were presented in the preceding section. The assessment of client capabilities, while not directly measured in all studies, are important outcome measures to be considered in reablement training or evaluation. In terms of assessing the efficacy of reablement programmes, and therefore informing the design of future programmes, client assessment outcomes will be presented in this capacity. In the Gerrish *et al*. (2017) study, all eight client participants were able to achieve successful medicines reablement by the end of their participation in the programme. In the Low *et al*. (2018) study, staff members who conducted the reablement programme noticed ‘positive changes in the units they worked in’, and described clients as more ‘settled’. 8). The data collected directly from the clients showed promising improvements in the clients’ mental health symptoms, functionality, and social-care related quality of life (Low *et al*., 2018). The Bergstrom *et al*. (2019) study based its outcome measures primarily on the Swedish version of the Canadian Occupational Performance Measure (COPM). The COPM measures the ‘self-assessed performance and satisfaction of valued activities in everyday life within the areas of self-care, productivity, and leisure’ (p. 6). Because this study was a feasibility study, no actual outcome measures were reported.

### Studies with no programme/assessment focus but useful to inform future reablement training programme development

Three of the studies included in this review (Gustafsson *et al*., 2019; Zingmark *et al*., 2019; Eliassen *et al*., 2020) are not focused on reablement training programmes or the evaluation of these programmes, but present information that could be useful to inform future reablement training programme design and development. The study by Eliassen *et al*. (2020) focused on how physiotherapy is performed in reablement settings and the content and delivery mode of this service. This study showed that reablement teams with flexible roles and division of labour were able to better meet the needs of clients than teams where roles were distinct and predefined (Eliassen *et al*., 2020). Gustafsson *et al*.’s (2019) study examined older adults’ perceptions of the components of successful short-term reablement care. The older adults found that a motivating caregiver who created a positive atmosphere, and who treated them with dignity and went ‘beyond the expected’ in terms of care delivered (Gustafsson *et al*., 2019:501) were the most important elements of successful reablement care. Zingmark *et al*.’s (2019) study documented the reablement practices of occupational (OT) and physical therapists (PTs) and found both key differences (e.g., duration of interventions) and important similarities (e.g., focus on mobility) in the way reablement activities are administered by these two sets of professionals. These findings illustrated the importance of critical reflection on the design of reablement intervention components and how OTs and PTs can best work together to promote a successful and positive reablement experience for clients (Zingmark *et al*., 2019).

## Discussion

This scoping review has demonstrated variation in the population, intervention type, theoretical framework, and outcome measures of included studies. These studies represent the range of settings where community service and health care organisations can deliver reablement programmes. Each training programme included in this review is initiated by an allied health professional or nurse and tailored to frontline staff with the aim for them to: better understood and apply the principles of reablement; support clients in reablement-orientated behaviour change (Stay Active at Home, ASSIST); build better relationships with clients; and increase knowledge and skills in reablement practices. Frameworks such as the ASSIST (OT) program assess changes in self-efficacy, perceived health, and well-being of the clients. The COPM is more focused on measures of self-assessed staff performance and client satisfaction with valued activities. Nursing frameworks include assessment of clients’ mental health symptoms, functionality, and social-care related quality of life. Qualitative measures of assessment focused on how staff can promote a positive and successful experience for clients. Consistent with the consensus definition of reablement (Metzelthin *et al*., 2020), these measures have been shown to contribute to improving and/or maintaining independence in individuals in a community setting through direct and collective assessments and interventions.

As a complex intervention, reablement has at its core a commitment to a person-centred, holistic approach with clients and the requirement for trained, interdisciplinary staff who are capable of conducting initial and ongoing assessment of clients. The studies in this review support this approach and highlight the importance of individual client assessment to support positive outcomes. The training programmes focused on reablement in this review also mostly draw from the model of person-centred care in different contexts, which inherently relies on individual client input and a collaborative approach to developing and assessment of activities included in individual plans. Theoretical underpinnings most commonly applied in the studies to the establishment of reablement principles were Bandura’s social learning theory of self-efficacy and behaviour change and Kotter’s eight-step model for change to facilitate a culture of assessment (Gorran Farkas, 2013; Montcalm, 1999).

In the studies reviewed, the application of these theories to practice provides the catalyst for ensuring the right environmental and cognitive factors are recognised as being important for human learning and behaviour and to bring about lasting change. As such they are pragmatic and philosophically aligned with reablement. Further exploration of these two approaches would have merit and advantage particularly where the researchers and training team work with the organisation to develop appropriately targeted learning and teaching resources, reflecting suitable reablement practices of working with clients to achieve goals (Maxwell *et al*., 2021).

Maxwell and colleagues (2021) found that post implementation of reablement practices there was evidence of improved motivation of staff which appeared to enhance morale and confidence. Important advantages of adopting a reablement ethos were also observed in other international studies (Rostgaard, 2018). Research from Denmark shows that staff working intensively with reablement experience a number of benefits (Rostgaard, 2018). Evidence indicates the importance of the relationship between care coordinators within organisations and direct care workers and that into the future this could be improved and developed to further enhance and deliver client care and reablement education within organisations (Maxwell *et al*., 2021). Improvements from the staff perspective included opportunities for regular meetings, seeking advice and discussing workforce-related and client-related issues. This is consistent with previous research (Rabiee & Glendinning 2011) that illustrates the features, content, and delivery of reablement services (such as a limited potential for staff to be independent, a lack of continuity of support and time pressures) may detract from its effectiveness in an organisation.

Furthermore, it has been suggested that the use of a pre-employment questionnaire to determine how commencing or potential employees might interact with clients and their families physically and emotionally may provide further evidence of supporting reablement in practice (Prior *et al*., 2020). A workforce aligned with the values of reablement in a community-based support field would promote the national aim of allowing people to age in place, improving client-centred care overall and will contribute to lessening the burden on residential aged care facilities (Prior *et al*., 2020).

## Limitations

The limitations in this study mainly relate to scope. Due to its exploratory nature, the study and the authors’ main interest were to establish the evidence associated with reablement practices in community settings. Other relevant studies conducted in hospital or rehabilitation settings therefor may have been excluded. There may have been a limitation from excluding studies before 2009; however, this is unlikely given that the framework of reablement has been introduced after that time.

## Conclusions

This review has identified that currently reablement programmes are not yet embedded as a framework for standard practice by community service providers in primary health care settings. Different programmes of training and client assessment are being designed based on single disciplinary approaches and the context in which they are delivered. Further developmental work is required to integrate the components of individual training programmes within interdisciplinary frameworks. This will achieve not only an integrated framework for delivery across settings but also develop an interdisciplinary workforce aligned with the values of reablement, promote ageing in place and ultimately lessen the burden on residential aged care facilities.

## Footnotes

1 Note that the Metzelthin et al. and Smeets et al. studies are referring to the same reablement programme (‘Stay Active at Home’).

## Supplementary material

For supplementary material accompanying this paper visit https://doi.org/10.1017/S1463423621000918. click here to view supplementary material
